# Dynamic subcellular localization of isoforms of the folate pathway enzyme serine hydroxymethyltransferase (SHMT) through the erythrocytic cycle of *Plasmodium falciparum*

**DOI:** 10.1186/1475-2875-9-351

**Published:** 2010-12-03

**Authors:** Martin Read, Ingrid B Müller, Sarah L Mitchell, Paul FG Sims, John E Hyde

**Affiliations:** 1 Manchester Interdisciplinary Biocentre, Faculty of Life Sciences, University of Manchester, 131 Princess Street, Manchester M1 7DN, UK; 2 Department of Biochemistry, Bernhard Nocht Institute for Tropical Medicine, Bernhard-Nocht-Strasse 74, 20359 Hamburg, Germany; 3 Western Comprehensive Local Research Network (WCLRN), University Hospitals Bristol NHS Foundation Trust, Upper Maudlin Street, Bristol, BS2 8AE, UK

## Abstract

**Background:**

The folate pathway enzyme serine hydroxymethyltransferase (SHMT) converts serine to glycine and 5,10-methylenetetrahydrofolate and is essential for the acquisition of one-carbon units for subsequent transfer reactions. 5,10-methylenetetrahydrofolate is used by thymidylate synthase to convert dUMP to dTMP for DNA synthesis. In *Plasmodium falciparum *an enzymatically functional SHMT (PfSHMTc) and a related, apparently inactive isoform (PfSHMTm) are found, encoded by different genes. Here, patterns of localization of the two isoforms during the parasite erythrocytic cycle are investigated.

**Methods:**

Polyclonal antibodies were raised to PfSHMTc and PfSHMTm, and, together with specific markers for the mitochondrion and apicoplast, were employed in quantitative confocal fluorescence microscopy of blood-stage parasites.

**Results:**

As well as the expected cytoplasmic occupancy of PfSHMTc during all stages, localization into the mitochondrion and apicoplast occurred in a stage-specific manner. Although early trophozoites lacked visible organellar PfSHMTc, a significant percentage of parasites showed such fluorescence during the mid-to-late trophozoite and schizont stages. In the case of the mitochondrion, the majority of parasites in these stages at any given time showed no marked PfSHMTc fluorescence, suggesting that its occupancy of this organelle is of limited duration. PfSHMTm showed a distinctly more pronounced mitochondrial location through most of the erythrocytic cycle and GFP-tagging of its N-terminal region confirmed the predicted presence of a mitochondrial signal sequence. Within the apicoplast, a majority of mitotic schizonts showed a marked concentration of PfSHMTc, whose localization in this organelle was less restricted than for the mitochondrion and persisted from the late trophozoite to the post-mitotic stages. PfSHMTm showed a broadly similar distribution across the cycle, but with a distinctive punctate accumulation towards the ends of elongating apicoplasts. In very late post-mitotic schizonts, both PfSHMTc and PfSHMTm were concentrated in the central region of the parasite that becomes the residual body on erythrocyte lysis and merozoite release.

**Conclusions:**

Both PfSHMTc and PfSHMTm show dynamic, stage-dependent localization among the different compartments of the parasite and sequence analysis suggests they may also reversibly associate with each other, a factor that may be critical to folate cofactor function, given the apparent lack of enzymic activity of PfSHMTm.

## Background

Malaria parasites are a major cause of mortality and morbidity, resulting in over a million deaths each year and 350 to 500 million clinically significant malaria infections [1]. Folate metabolism is the target of a number of anti-malarial drugs, which, though compromised by the occurrence and spread of resistance within parasite populations, remain important in treatment and prophylaxis [2,3]. For almost all organisms, the folate pathway is essential in maintaining a constant supply of cofactors that act as donors or acceptors of one-carbon (C_1_) units in a variety of biosyntheses. In malaria parasites, the most prominent of these is the synthesis of pyrimidines required for DNA replication [4]. Unlike mammals, *Plasmodium falciparum *cannot salvage thymidine and thus relies completely on the folate-dependent production of dTMP. The folate pathway can be conveniently divided into two main sections: the first five enzyme activities effect the *de novo *biosynthesis of the basic folate moiety, 7,8-dihydrofolate (DHF), with further enzymes interconverting the fully reduced form 5,6,7,8-tetrahydrofolate (THF) to the various derivatives utilized in C_1 _transfer reactions. Plants and most micro-organisms, including many protozoa, are able to synthesize folates *de novo*. In contrast, higher organisms must obtain folate from the diet or commensal microorganisms. It has been shown that *P. falciparum *has the ability to exploit both *de *novo synthesis and folate salvage routes for its metabolic needs [5-7].

The later part of the folate pathway directly relevant to DNA replication is termed the thymidylate cycle. In this, dihydrofolate reductase (DHFR; EC 1.5.1.3) catalyses the reduction of DHF to THF. Serine hydroxymethyltransferase (SHMT; EC 2.1.2.1), the subject of this study, reversibly catalyses the conversion of serine to glycine, whereby the hydroxymethyl group of the former is transferred to THF yielding 5,10-methylenetetrahydrofolate (5,10-methylene-THF), which is then used by thymidylate synthase (TS; EC 2.1.1.45) as the C_1 _donor to convert dUMP to dTMP. Concomitantly, the folate cofactor is oxidized to the dihydro-form, making a functional cycle that is capable of reducing this back to THF essential for continued DNA synthesis. A further activity, folylpolyglutamate synthase (FPGS; EC 6.3.2.17), part of a bifunctional protein also carrying dihydrofolate synthase (DHFS; EC 6.3.2.12) [8-10] adds a variable length polyglutamate tail to reduced folate cofactors, a phenomenon involved in subcellular storage and the retention of folates within the cell [11-13].

Despite much research detailing the biochemistry of the folate pathway and the genetic basis of resistance to antifolate drugs, there has been very little investigation of the subcellular location of folate pathway enzymes or their metabolites in malaria parasites. In other eukaryotes there is substantial evidence for the compartmentalization of folate metabolism within the cell. In particular, fully reduced substituted folates (such as 5-methyl-THF) appear not to exchange between mitochondrial and cytoplasmic compartments, suggesting that limited transport of intermediates between subcellular compartments may be an important factor in enzyme localization [14].

Consistent with this, the majority of methyl derivative forms are associated with the vacuole and cytosol, whereas formyl derivatives make up the greatest proportion of folates located within organelles, at least in plants [15]. In these organisms, the first two enzymes of biosynthesis, GTP cyclohydrolase I (GTPCH; EC 3.5.4.16) and dihydroneopterin aldolase (DHNA; EC 4.1.2.25), are found exclusively in the cytoplasm, whereas most of the remaining enzymes are located exclusively in the lumen of the mitochondrion. SHMT and FPGS are exceptional as they are found in both the cytoplasm and mitochondrion as well as the plant chloroplast. The two forms of SHMT, SHMTc and SHMTm, occur as distinct proteins encoded by different genes [16], but the chloroplast enzyme appears not yet to have been characterized as a separate isoform. Distinct SHMT isoforms are also found in the cytoplasm and mitochondria of yeast and the trypanosomatid *Leishmania major *[17], and in mammals, the distribution of both SHMTc and SHMTm differs between tissues and at different stages of development [18,19]. Glycine formed from SHMT acting on serine can feed into the glycine cleavage complex (GCV) of the mitochondrion, which provides an additional source of C_1 _units by transferring the α-carbon of glycine onto THF [20]. *Plasmodium falciparum *SHMT, described here as PfSHMTc, is encoded by a single copy gene at locus PFL1720w [8] and its enzymic properties are well characterized [21-23]. A second open reading frame has also been identified (PF14_0534) that encodes a product with an 18% identity to PfSHMTc and incorporates a putative mitochondrion-specific tag [24]; this protein is described here as PfSHMTm. However, its sequence displays an almost complete lack of conservation of those amino acids that constitute the active site residues of all other SHMT isoforms, both cytoplasmic and mitochondrial, consistent with a failure to detect SHMT activity in a recombinant form of the protein [23]. The metabolic or other function of this related gene product thus remains to be identified, and particularly whether it could act in conjunction with a GCV in plasmodial mitochondria. Components of a potential GCV have been identified bioinformatically in *P. falciparum *[24], although experimental evidence for their mitochondrial location has thus far only been established for the H-protein [25].

The multiplicity of environments that the parasite must accommodate in its complex life cycle suggests that adaptability in folate metabolism and its enzymes is highly probable, and that variation in enzyme localization over the life cycle might occur. Additionally the parasite exhibits a number of unusual developmental features that could result in differences in folate metabolism from other eukaryotes. The schizogonic nature of asexual reproduction, with its repeated and apparently asynchronous cryptomitoses, results in an atypical cell cycle [26,27]. Peculiarities in the timing and duration of events associated with DNA replication may result in temporal variation in the demand for pyrimidine synthesis [28]. Here, patterns of localization of PfSHMTc during the erythrocytic cycle of *P. falciparum *are investigated because of its key role in dTMP synthesis, the strongly modulated level of transcriptional control of its gene and its relatively higher levels of expression compared to other enzymes in the folate pathway [29-31]. In parallel, the localization of the enigmatic PfSHMTm protein is investigated, which shows similarities in its behaviour, but with distinct and important differences from the PfSHMTc isoform.

## Methods

### Cloning and heterologous expression of the *pfshmt *genes

The full-length cytoplasmic *pfshmt *gene (PFL1720w) was amplified from cDNA previously cloned into pMALc2x (New England Biolabs). The intronless full-length *shmt *homologue PF14_0534 was amplified from K1 isolate genomic DNA, and both products were cloned into the pET-46 Ek/LIC vector (Novagen). The cytoplasmic *pfshmt *clone was expressed in the BL21 (DE3) pLysS expression host whilst the PF14_0534 ORF was expressed in Rosetta 2 (DE3) pLysS (Novagen). Cultures of both clones were harvested using the Bugbuster kit (Novagen) and the insoluble phases subjected to SDS-PAGE and subsequently blotted onto nitrocellulose. Fractions were loaded on the PAGE gel to give equal protein quantities in the bands of interest between the two expressed protein products. Western blots were probed with anti-PfSHMTc IgY or anti-PfSHMTm IgY, or anti-polyhistidine IgG primary antibodies (see below) followed by the appropriate AP conjugate secondary antibodies (Promega), and developed using standard methods [32].

### Parasite culture and transfection

Parasites (either K1 or 3D7) were grown in 25 cm^2 ^tissue culture flasks with 1 ml of blood (type O; 50% haematocrit) and 10 ml of medium as described [33]. Flasks were harvested at a parasitaemia of 8 - 15%. The use of synchronous cultures was investigated but yielded no significant advantage owing to the inherent asynchrony of the repeated mitoses in individual cells [26,34]. The developmental stage of a particular parasite within asynchronous cultures was ascertained through its size, haemozoin development, number of nuclei and overall morphology. For GFP-labelling studies, 3D7 parasites were transfected with appropriate plasmid constructs encoding SHMT-GFP fusion proteins essentially as described [35], using the primers pfSHMTm-kpn-s (gcgcggtaccATGCTGAAGGAGTTTGTTAAAAATG) and pfSHMTm100-avr-as (gagacctaggGCAACCCCAATATTTCTTTTGTAA) to clone the truncated *pfshmtm *gene described in Results into the pARL1a- vector [36].

### Western blotting of parasite extracts

Parasite extracts were also prepared for western blotting by freeze-thawing, in which 1 ml of blood at 50% haematocrit and *ca*. 10% parasitaemia was saponin lysed and washed in PBS. The resulting parasite pellet was resuspended in 0.1 ml of deionized water and subjected to 5 rounds of freezing and thawing. Following centrifugation the supernatant was recovered and 20 μl (equivalent to *ca*. 10^8 ^parasites) used per lane on 12% acrylamide SDS-PAGE gels. Protein was transferred to nitrocellulose using a Biorad Mini Protean II blotter; blots were probed with primary antibody and secondary alkaline phosphatase-conjugated antibodies (Promega).

### Antibodies

The PfSHMTc primary polyclonal antibody (IgY) was raised in chickens against the denatured product of a 70-codon DNA segment [369-GIRIG...QWAKN-438] located towards the 3' terminus of the *pfshmt *gene (PFL1720w) expressed in *E. coli *as a GST fusion. The PfSHMTm primary polyclonal antibody (IgY) was raised in chickens against the denatured full-length gene product expressed in *E. coli *as a His-tagged fusion. The same gene product was additionally used to raise antibodies (IgG) in rabbits. All three antibodies were commercially produced by Eurogentec. The donated apicoplast-specific antibody, anti-acyl carrier protein (anti-ACP; IgG) was raised in rabbits [37], as were the donated antibodies against the cytoplasmic enzymes chorismate synthase and cyclin-dependent protein kinase 5 [38]. The donated 3D7 parasite transfectant strain carrying pSSPF2/PfACP-DsRED [39] was used to confirm data obtained with the anti-PfACP antibody and control for possible interactions between this and other primary antibodies used simultaneously. The secondary antibodies, Alexafluor (488, 546 and 594 nm) goat anti-chicken IgY and anti-rabbit IgG, were obtained from Molecular Probes, as were the MitoTracker Orange CMTMRos mitochondrial probe and the DNA stain YOYO-1 (491/509 nm).

### Immunofluorescence: parasite fixation, permeabilization and staining

A preparative method was developed to maximize the fluorescence intensity of the target proteins by ensuring a high degree of penetration of both primary and secondary antibodies and sufficient incubation with the primary antibody. This was necessary as soluble enzymes are frequently found in relatively low concentrations and are thus less readily visualized than structural, membrane-associated or exclusively organelle-bound proteins. Moreover, malarial folate pathway enzymes are known from both transcriptional and proteomic measurements to be expressed at low levels [29-31]. Preservation of the erythrocyte membrane proved to be largely impracticable due to lysis caused by a combination of detergent extraction and the mechanical stresses inherent in the mixing and centrifugation steps. Transmission light images were thus relatively poor and often obscured by erythrocyte ghosts; they are included merely to indicate the position of the haemozoin within the parasite pigment vacuole. However, the high degree of preservation of the parasites themselves using this method is evident in the undistorted images of internal fine structure shown. Significantly, the control apicoplast-specific antibody (anti-PfACP; see below) showed no fluorescence within the apicoplast in the absence of detergent permeabilization, demonstrating that any immunofluorescence investigation of the internal distribution of proteins within parasites should always ascertain the necessity of such a step.

Giemsa-stained thin blood smears were taken from all cultures used for immunofluorescence imaging upon harvesting to ensure that parasites showed normal undamaged morphology and healthy growth. Cultures selected for mitochondrial staining were incubated at 37°C for 45 min with MitoTracker Orange CMTMRos freshly dissolved in dimethylsulfoxide to give 100 nM final concentration in the medium. A 1 ml volume of parasitized erythrocytes was harvested by centrifugation (3,000 g, 5 min). Pelleted cells were resuspended and fixed in 5 ml freshly prepared 3.7% (w/v) paraformaldehyde in phosphate buffered saline (PBS) for a minimum of 2 h at 4ËšC. Following fixation, the parasitized blood was centrifuged (as above) and washed twice in blocking/wash solution (1 ml PBS, 0.5% (w/v) BSA, 2% (v/v) bovine serum; Sigma) in parallel-sided, screw-capped microfuge tubes with rotational mixing at room temperature for 5 min followed by centrifugation (8,000 g, 30 s). Cells were then incubated in wash buffer plus 0.25% (v/v) Triton X-100 for 5 min to increase permeability. After a further three washes in wash buffer (also used in all subsequent washes), primary antibodies (diluted 1:100 in wash buffer) were added and incubated overnight at 4°C. The samples were then washed four times and incubated with fluorescent secondary antibodies, diluted to the manufacturer's specification (usually 2.5 μl in 1,000 μl), for 2 - 4 h at room temperature. This was followed by three washes, then the DNA was labelled by the addition to the cells suspended in 1 ml of wash solution of 20 μl YOYO-1 (diluted 1:1,000 in wash buffer) and incubation at room temperature for 5 min. The cells were then centrifuged, washed for 1 min and then immediately centrifuged again (as above). The pelleted cells were resuspended in 100 - 250 μl Mowiol (Harco, UK) mountant and mounted on microscope slides under a coverslip [32].

### Microscopy

Parasites labelled for immunofluorescence were viewed by laser scanning confocal microscopy using a Zeiss Axiovert 200 M microscope with argon (548-514 nm) and helium/neon lasers (543 nm, 632.8 nm) using a 100× oil immersion objective lens. Images were viewed and analysed using a combination of Zeiss LSM image software and Imaris 5.7.1 software (Bitplane Scientific Solutions). The latter allows the qualitative display of combined colours from co-localized probes to be quantitatively analysed by providing measurements of their overlap in three dimensions by analysing z-stack scans taken through the whole span of the organelle [40]. This enables a much more accurate assessment of coincidence of the labels than is possible with single-plane images. Co-localization is expressed as a percentage of the individual fluorochrome volume and material (the latter derived from volume and fluorescence intensity) that occupies the same 'voxels' (three-dimensional pixels) as the second fluorochrome. For a single cell, similar values between volume % and material % co-localized indicate similar concentrations inside and outside the organelle, whereas a higher organellar material % compared to volume % indicates concentration of the target protein relative to the cytoplasm. Three-dimensional projections were created from scans with a z-axis interval of 0.2 μm. This was the minimum increment possible before the scans became excessively long, resulting in unacceptable levels of photobleaching. Scans were sequential, with each colour wavelength scanned in rotation for each single plane image or within each plane in a z stack. For clarity, orange wavelength fluorescence (Alexafluor 546, DsRED and MitoTracker) is false-coloured green, green wavelength fluorescence (Alexafluor 488 and YOYO-1) is false-coloured blue, but far-red fluorescence (Alexafluor 594) is unchanged in all of the images displayed. Transfected parasites expressing GFP-fusion protein endogenously were imaged as previously described using Hoechst 33342 to visualize the nuclei [35] and MitoTracker Red CMXRos (0.625 nM) to visualize the mitochondrion. All images presented are representative examples of each feature as seen in multiple samples.

### Imaging controls

Antibody extracts from pre-immune yolk showed no bands when applied to western blots of total parasite proteins in parallel with the antigen-specific antibodies. Control slides labelled with secondary antibodies alone, or with combinations of secondary antibodies (used to control for artifactual interactions), showed no visible fluorescence when scanned using identical microscope settings and computer processing parameters as those used in producing the images shown. As the anti-PfSHMTc antibody was raised to a GST fusion polypeptide, the possibility of it recognising a plasmodial GST orthologue was excluded using a commercial polyclonal anti-GST IgG (GE Healthcare) on parasites as described above, which also showed no visible fluorescence. Haemozoin auto-fluoresces at a number of wavelengths, however its crystalline nature makes its fluorescence easily recognized and an appropriate choice of filters avoided interference with any of fluorochromes used.

To provide controls against the possibility that the preparative method used might artifactually produce organellar fluorescence in a non-specific manner, polyclonal antibodies against two unrelated enzymes, chorismate synthase and cyclin-dependent protein kinase 5, were also employed, which had been previously characterized as showing a simple cytoplasmic distribution in *P. falciparum *[38]. These control antibodies were employed with 3D7 parasites expressing the DsRED labelled apicoplast-specific protein PfACP [39]. Parasites were treated with control antibody in parallel procedures alongside parasites treated with anti-PfSHMTc and anti-PfSHMTm antibodies. No level of apicoplast-specific fluorescence was observed with either control antibody, which produced a generalized staining of the parasites with no evidence of fluorescence adopting the shape of apicoplasts (see Additional file 1 Negative control images for organellar staining). Furthermore, to exclude the possibility that artifactual interactions between the apicoplast-specific antibody anti-PfACP and the anti-PfSHMT antibodies were occurring, the latter were also used in conjunction with the above DsRED-transfected parasites, yielding identical patterns as those obtained using two primary antibodies simultaneously (see Results).

## Results

### Antibody specificity with respect to PfSHMTc and PfSHMTm

**Figure 1:**
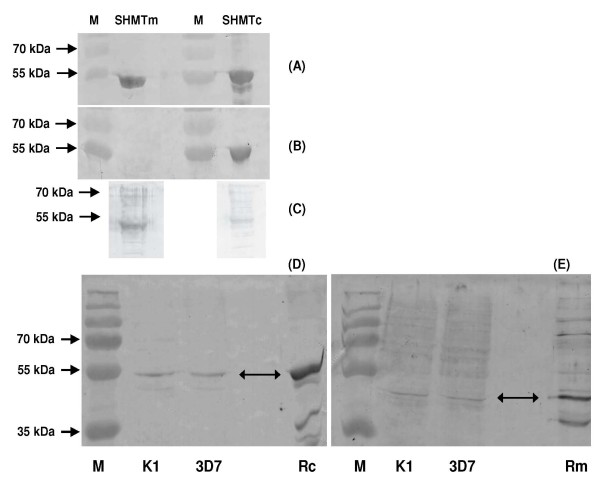
**Specificity of the polyclonal anti-PfSHMT preparations**. Full-length His-tagged recombinant protein (500 ng) expressed from the genes encoding PfSHMTc (PFL1720w) and PfSHMTm (Pf14_0534) probed on western blots with (A) anti-polyhistidine IgG, (B) the anti-PfSHMTc and (C) the anti-PfSHMTm preparations used for subsequent immunofluorescence studies. Panels D and E are western blots of total parasite extracts from K1 and 3D7 probed with anti-PfSHMTc (D) and anti-PfSHMTm (E). Rc, recombinant PfSHMTc; Rm, recombinant PfSHMTm; M, prestained molecular weight markers.

The *pfshmt *gene from *P. falciparum *(PFL1720w) [41] encodes a product that has been functionally characterized as a conventional cytoplasmic SHMT [21-23]. However, a predicted SHMT-like gene product (PfSHMTm, encoded on PF14_ 0534) was also identified that carries a putative mitochondrial signal sequence [24] with 18% amino acid identity and 44% similarity to PfSHMTc, but lacks almost all (16 of 21) of the known, very highly conserved residues [42] contributing to the active site in SHMT orthologues from other organisms, whether cytoplasmic or organellar (See Additional file 2 Sequence alignments of the PfSHMT isoforms). Despite the relatively low level of identity, it was essential to establish the specificity of the anti-PfSHMTc and anti-PfSHMTm antibodies that had been raised to be certain of the identity of the protein yielding positive signals. Both full-length open reading frames were therefore cloned in *Escherichia coli *expression systems and equal amounts of protein products processed for western blotting. The anti-PfSHMTc antibody recognized the heterologously expressed cognate protein (Figure 1B) and blots of total parasite lysates from two lines, K1 and 3D7, showed a single band also at the predicted size (49.8 kDa) for the full length PfSHMTc protein (Figure 1D). Importantly, there was no evidence for cross-reaction with the PfSHMTm product of PF14_0534 (Figure 1B), whereas control anti-His-tag antibodies recognized both recombinant products essentially equally (Figure 1A). This engendered confidence that subsequent immunofluorescence signals using the cognate antibody arose solely from PfSHMTc. In the case of PfSHMTm, this antibody was raised to the whole protein (unlike the anti-PfSHMTc antibody), some cross-reaction with PfSHMTc was not unexpected and was evident on blots against recombinant protein. However, this was approximately fourfold less intense than that seen in recognising the cognate PfSHMTm protein (Figure 1C). Against parasite extracts, the anti-PfSHMTm antibodies gave a predominant band with the same mobility as the recombinant protein (Figure 1E). It was noted on all blots that PfSHMTm ran slightly ahead of PfSHMTc, despite its somewhat higher predicted molecular weight (55.2 kDa). These differences in specificity led us to conclude that the differences seen below in immunofluorescence images of parasites probed with anti-PfSHMTc from those produced using anti-PfSHMTm are a reliable indicator of biologically significant variations in the distribution of the respective target proteins.

### Cytoplasmic distribution of the PfSHMT isoforms

**Figure 2:**
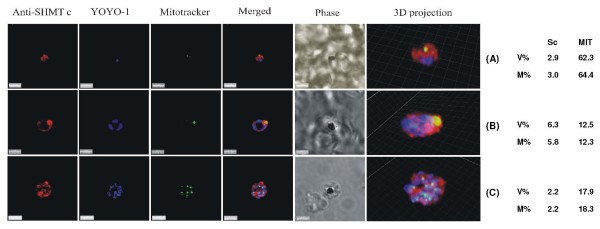
**PfSHMTc immunofluorescence images showing localization in the mitochondrion**. (A) Mid-trophozoite showing the association of a small mitochondrion with PfSHMTc fluorescence. (B) Early schizont showing association of an enlarged globular mitochondrion with a region of more intense PfSHMTc fluorescence. (C) Late schizont showing very little co-localization of mitochondria with areas of PfSHMTc fluorescence. Mitochondria are closely aligned to nuclei and show some co-localization with YOYO1 staining (scale bars 3 μm). The associated table shows the percentage volume (V%) and material (M%) co-localization data for PfSHMTc (Sc) and MitoTracker (MIT) fluorescence.

**Figure 3:**
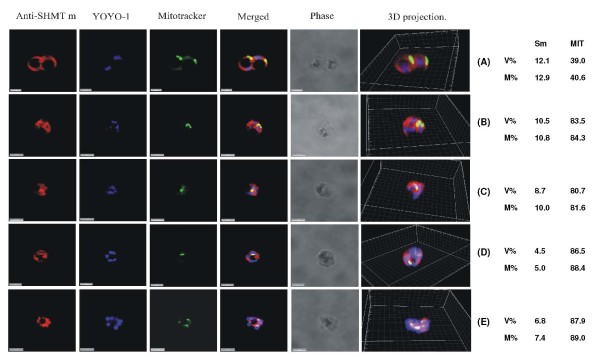
**PfSHMTm immunofluorescence images showing localization in the mitochondrion**. (A) Two late trophozoites. (B-D) Mitotic schizonts. (E) Post-mitotic schizont. The images show the persistence of co-localization of PfSHMTm fluorescence with the mitochondria throughout the developmental cycle (scale bars 3 μm). The associated table shows the percentage volume (V%) and material (M%) co-localization data for PfSHMTm (Sm) and MitoTracker (MIT) fluorescence.

**Figure 4:**
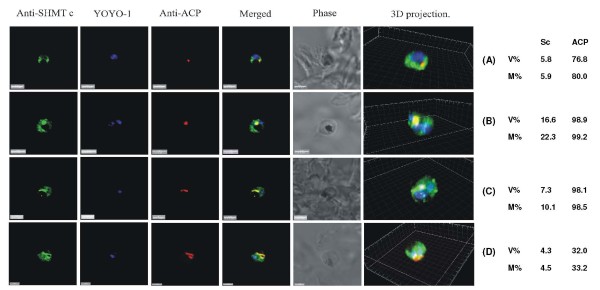
**PfSHMTc immunofluorescence images showing localization in the apicoplast**. (A) Mid-trophozoite showing the co-localization of plastid specific fluorescence with PfSHMTc fluorescence. (B) Early mitotic schizont showing very marked co-localization of plastid specific fluorescence (enlarged globular apicoplast) with PfSHMTc fluorescence. (C) Mitotic schizont showing very marked co-localization of plastid specific fluorescence with PfSHMTc fluorescence. The plastid here is in the early stages of elongation. Note also the small punctate concentration of PfSHMTc fluorescence on the periphery of the unstained region of the parasite corresponding to the pigment vacuole. (D) Mitotic schizont developmentally a little later than (C) showing a mitochondrion in the early stages of ramification. The area of intense PfSHMTc fluorescence follows the 'Y' shape of the mitochondrion closely (scale bars 3 μm, except (D) which is 2 μm). The associated table shows the percentage volume (V%) and material (M%) co-localization data for PfSHMTc (Sc) and acyl carrier protein (ACP) fluorescence.

**Figure 5:**
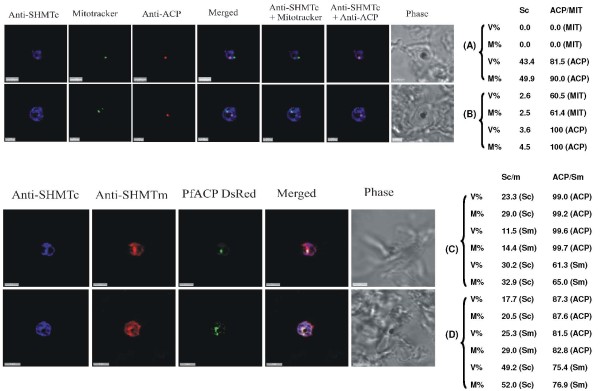
**Triple-labelling experiments**. (A) and (B) Combined mitochondrial and apicoplast images probed with anti-PfSHMTc. These do not show nuclear morphology, therefore the erythrocytic cycle stage cannot be precisely ascertained; however, the size of the organelles and overall size of the parasites in (A) and (B) suggest that both are mid trophozoites. In (A) the parasite is probed with anti-PfSHMTc, MitoTracker and anti-ACP (plastid). The plastid is coincident with an area of marked PfSHMTc fluorescence, whereas the mitochondrion shows no evidence of coincident PfSHMTc fluorescence. In (B) the parasite is probed with anti-PfSHMTc, MitoTracker and anti-ACP (plastid). The plastid is coincident with a discrete area of PfSHMTc fluorescence, whereas the mitochondrion is located in a pocket of lower PfSHMTc fluorescence. (C) Parasite is probably a late trophozoite and (D) a mitotic schizont. Both parasites were expressing DsRED-labelled ACP and were probed with both anti-PcSHMTc (IgY) and anti-PfSHMTm (IgG). The distribution of the two SHMT fluorescence signals are similar but not identical, and both co-localize with the apicoplast (scale bars (A) and (C), 3 μm, (B) 2 μm, (D) 4 μm). The associated table shows the percentage volume (V%) and material (M%) co-localization data for PfSHMTc (Sc), PfSHMTm (Sm), MitoTracker (MIT) and acyl carrier protein (ACP) fluorescence.

**Figure 6:**
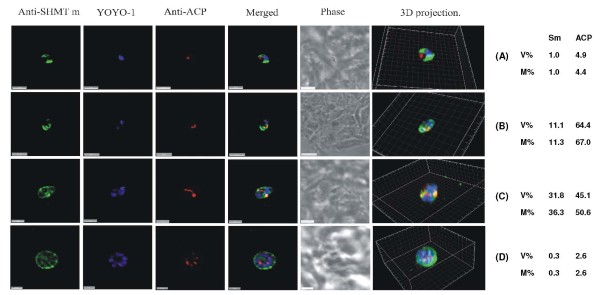
**PfSHMTm immunofluorescence images showing localization in the apicoplast**. (A) Early trophozoite showing no apicoplast PfSHMTm co-localization. (B) Early mitotic schizont with PfSHMTm fluorescence conforming closely to the 'C' shaped apicoplast. (C) Mitotic schizont with an elongating apicoplast, PfSHMTm fluorescence is concentrated within the distal portions of the organelle with little fluorescence in the medial section. (D) Post-mitotic schizont showing little spatial coincidence of PfSHMTm and the multiple apicoplasts (scale bars 3 μm, except (D). which is 2 μm). The associated table shows the percentage volume (V%) and material (M%) co-localization data for PfSHMTm (Sm) and acyl carrier protein (ACP) fluorescence.

**Figure 7:**
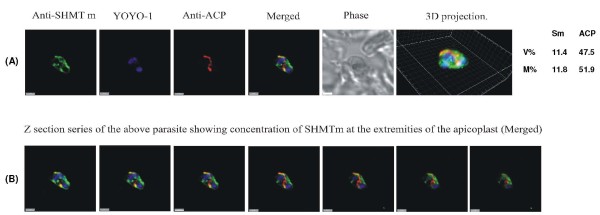
**PfSHMTm apicoplast immunofluorescent images illustrating the concentration of fluorescence in the extremities of elongating apicoplasts**. (A) Mitotic schizont with an elongating apicoplast. (B) A z-stack series with an interval of 0.2 μm through the same parasite showing the concentration of PfSHMTm fluorescence in the distal portions of the apicoplast and the relative lack of PfSHMTm fluorescence in its medial section (scale bars 2 μm).

**Figure 8:**
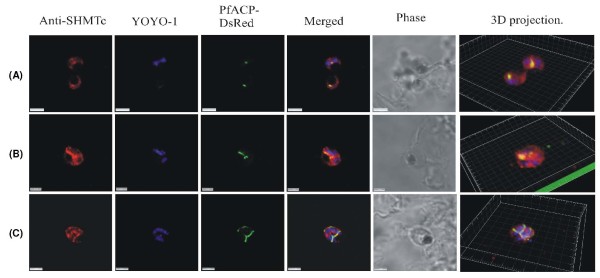
**Positive control images using endogenously expressed DsRED-tagged ACP instead of anti-ACP antibodies**. The use of only one primary antibody, anti-PfSHMTc, with expressed DsRED tagged Pf ACP, was aimed at eliminating any possibility of artifactual fluorescence arising from interactions between two primary antibodies used simultaneously. (A) Two parasites, upper parasite is undergoing its first division, lower parasite is a late trophozoite. (B) Mitotic schizont with elongating apicoplast. (C) Mitotic schizont with ramifying apicoplast. All parasites show co-localization of anti-PfSHMTc fluorescence with the apicoplast, closely following the shape of the organelle, identical results to those obtained using two primary antibodies (scale bars (A) and (C) 3 μm, (B) 2 μm).

**Figure 9:**
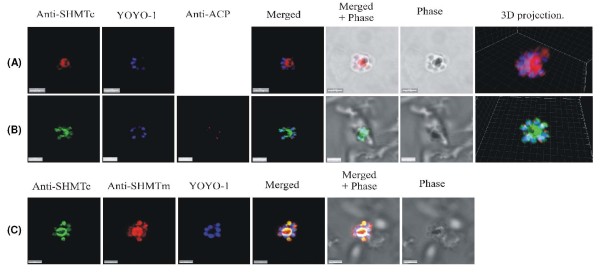
**Late schizonts show a central concentration of PfSHMTc fluorescence**. (A) Post-mitotic schizont showing a concentration of PfSHMTc fluorescence in the centre of the parasite, and overlapping the outer zone of haemozoin. PfSHMTc is largely excluded from the nuclei. (B) Post-mitotic schizont showing a concentration of PfSHMTc fluorescence in the centre of the parasite as well as at low intensity in the multiple small apicoplasts. Note the merozoite buds arranged in a radial pattern centred on the future residual body. (C) A post-mitotic parasite probed with both anti-PfSHMTc (IgY) and anti-PfSHMTm (IgG). Both SHMT proteins show a similar, but not identical distribution, as described for image series (A) and (B) above (scale bars 3 μm).

SHMT subcellular distribution in a number of organisms shows a partition between cytoplasmic SHMT and distinct isoforms of the enzyme located within organelles. As only PfSHMTc has thus far been confirmed as enzymatically active in *P. falciparum *[21-23], a single cellular location might be predicted. However, initial probing using its cognate antibody showed that PfSHMTc does not follow such a simple distribution pattern during the erythrocytic cycle. All stages showed an expected generalized cytoplasmic staining and this, by visual examination and volumetric analysis by the Imaris software, is where the majority of the PfSHMTc molecules are located for most of the time. However, fluorescence brightness within the cytoplasm was not uniform and constriction of cytoplasm between organelles, especially nuclei, produced a patchy appearance (Figure 2). The PfSHMTm protein (Figure 3) showed an almost identical cytoplasmic distribution to that described for the PfSHMTc enzyme, as can also be seen in Figure 4, 5, 6, 7, 8 and 9, in which the anti-PfSHMTc and anti-PfSHMTm antibodies are used in various combinations with organellar labels. However, images obtained where both antibodies were used in combination did show some minor differentiation in cytoplasmic localization and relative concentration within individual parasites, exemplified by Figure 5C, D and 5F.

### Mitochondrial localization of PfSHMTc

The mitochondrion and the apicoplast undergo a similar, though not simultaneous, morphological evolution during the development of erythrocytic stage parasites. The two organelles are found in close physical association and a junction between their respective membranes has been described [43,44]. The organelles increase in size, and in the case of the K1 isolate used here, were often observed to adopt a globular shape in the early schizont stage; thereafter they lengthen and ramify, eventually dividing to allow one of each organelle to associate with each individual developing merozoite [45]. These organelles thus have a requirement for folate pathway metabolites for the synthesis of DNA precursors needed for the replication of their genomes.

The mitochondria, visualized using MitoTracker, showed some evidence of associated PfSHMTc fluorescence throughout the erythrocytic cycle but predominantly during the stages associated with DNA replication. In many early and late parasites, the mitochondria were physically very small and consequently it could not be concluded with any certainty that PfSHMTc fluorescence was within the organelle lumen or merely in the adjacent cytoplasm. Indeed some early to mid-trophozoites showed no evidence of PfSHMTc fluorescence within their mitochondria. However, some mid-trophozoites showed a more convincing co-localization, e.g. Figure 2A, while the larger mitochondria found in very late trophozoites and early schizonts, such as shown in Figure 2B, clearly showed PfSHMTc fluorescence within the lumen, though at a similar concentration to that in the immediately surrounding cytoplasm. Figure 2C is an example of a post-mitotic schizont where very little co-localization remains.

Calculation of the levels of co-localization of PfSHMTc and MitoTracker reinforces this qualitative conclusion. The percentage of PfSHMTc material co-localizing varied between 2.2% and 5.8% (Figure 2) and the percentage volume of PfSHMTc co-localized showed only a similar, or slightly higher, value in comparison, confirming that the mitochondrion does not accumulate a noticeably higher concentration of PfSHMTc than that found in the cytoplasm. The three-dimensional projection within Figure 2C gives a particularly good view of a post-mitotic schizont showing the close spatial connection between the nuclei and mitochondria destined to occupy the same daughter merozoite. However, the mitochondria in this late stage parasite showed little evidence of PfSHMTc staining.

### Mitochondrial localization of PfSHMTm

The use of anti-PfSHMTm revealed a different pattern of mitochondrial co-localization. The PfSHMTm protein, in contrast to PfSHMTc, was found strongly associated with the mitochondria throughout the erythrocytic cycle, from early trophozoites to late, post-mitotic, schizonts. The mitochondrion was always found within regions of relatively high intensity PfSHMTm fluorescence (Figure 3A and 3E) and in many instances the shape of the PfSHMTm fluorescence conformed to the shape of mitochondria (Figure 3B, C and 3D). Importantly, there were no instances of scanned images where mitochondria were found without associated PfSHMTm fluorescence or where such fluorescence was visibly lower than that of the adjacent cytoplasm. However, the quantitative analysis for Figure 3 gave very similar figures for percentage PfSHMTm material co-localized with the MitoTracker compared with percentage volume co-localized, suggesting that there was no active accumulation of PfSHMTm within the mitochondria above the levels in the cytoplasm. The percentages of PfSHMTm material co-localized with MitoTracker varied between 5.0% and 12.9%, a higher range of values than measured for PfSHMTc (2.2 - 5.8%).

### Apicoplast localization of PfSHMTc

In contrast to the relatively weak spatial association between subcellular PfSHMTc distribution and the mitochondrion, the apicoplast exhibited a distinctly more pronounced relationship. The apicoplast was visualized in two ways: using antibodies to acyl carrier protein (anti-ACP), which is apicoplast specific [37,45] and using a transfected 3D7 line constitutively expressing DsRED-tagged PfACP [39]. The parasite shown in Figure 4A was at the mid-trophozoite stage, and although the apicoplast was still relatively small, PfSHMTc fluorescence was clearly co-localized with anti-ACP, indicating that it was within the lumen of this organelle. The parasites in Figure 4B and 4C are early schizonts, at which stage the apicoplast is considerably larger in absolute volume, as well as relative to overall cell volume. In the K1 isolate used in these images, the apicoplast often assumes first an enlarged globular form, which then elongates before ramifying. All of these parasites showed a bright PfSHMTc fluorescence coincident, or largely coincident, with the anti-ACP fluorescence that defines the position of the apicoplast, with the surrounding general cytoplasmic PfSHMTc fluorescence being perceptibly less bright. The parasite shown in Figure 4B displays the earlier globular apicoplast morphology, the parasite in Figure 4C contains an apicoplast that has started to elongate. The three-dimensional projection of the parasite in Figure 4C also allows a clear visualization of the small punctate concentrations of fluorescence that are suggestive of a vesicle-associated location of PfSHMTc, often seen in close proximity to the haemozoin containing pigment vacuole in trophozoite and early schizont stages. The parasite in Figure 4D shows an apicoplast in the ramifying stage of its development and the correspondence of the anti-PfSHMTc fluorescence to the 'Y' shaped apicoplast is striking. In the 3D7 transfectant expressing PfACP with a DsRED tag, the development of the apicoplast did not exhibit the globular stage often seen in K1 parasites, with narrow ramifying apicoplasts being far more evident (Figure 8B and 8C). However, the close coincidence of the apicoplast and PfSHMTc fluorescence was equally evident as when using K1 and two primary antibodies (see also below).

Quantitative analysis again supports the visual interpretation of the apicoplast data. The trophozoite shown in Figure 4A had a percentage material co-localization of PfSHMTc with anti-ACP of 5.9% and a percentage volume co-localization of 5.8%, indicating that the PfSHMTc fluorescence in this parasite was not appreciably higher within the apicoplast than without. The early schizont stage parasites in Figure 4B and 4C showed significantly higher percentages of PfSHMTc material co-localization of 10.1% and 22.3%, indicating that a considerable proportion of the PfSHMTc of these particular parasites was located within the comparatively small volume of the apicoplast. Moreover, the percentage material co-localized for PfSHMTc fluorescence in these two parasites was about one-third higher than the respective percentage volumes, reinforcing the visual impression that in these parasites PfSHMTc was at a higher concentration within the apicoplast than in the cytoplasm generally. A slightly later parasite (Figure 4D), showing a ramifying apicoplast, displayed a somewhat lower level of co-localization of PfSHMTc with anti-ACP of 4.5% at a concentration that is again no higher than that of the surrounding cytoplasm.

A direct comparison between mitochondrial and apicoplast PfSHMTc concentrations was made in triple staining experiments. In this case, the limitations of wavelengths available precluded using a dye to simultaneously stain the DNA so that the precise stage of the parasites viewed was not clearly discernible; however, the size of the organelles and overall size of the parasites suggest that those shown in Figure 5A and 5B are mid-trophozoites. In these experiments, PfSHMTc was stained using Alexafluor anti-chicken IgY 488 nm (false coloured blue), which proved to be especially prone to bleaching and therefore unsuited to the repeated exposure to laser light necessary in building a z-stack scan. Unlike the other images presented here, therefore, those showing both the mitochondrion and the apicoplast are from single plane scans where both organelles were in the same z-axis plane. The parasite in Figure 5A shows apicoplast-specific fluorescence located within a discrete region of bright PfSHMTc fluorescence, whereas in contrast, the mitochondrion appears to have no associated PfSHMTc fluorescence. The parasite in Figure 5B also shows the apicoplast fluorescence within a region of high PfSHMTc fluorescence whilst the mitochondrion occupies a pocket of lower intensity PfSHMTc fluorescence. Quantitative analysis confirmed the much more substantial association of PfSHMTc with the apicoplast than with the mitochondrion. As these figures refer to pixels in a single plane rather than voxels in a three-dimensional projection from a z-stack scan, extrapolation to volumetric values was unsafe in this particular case.

### Apicoplast localization of PfSHMTm

**Figure 10:**
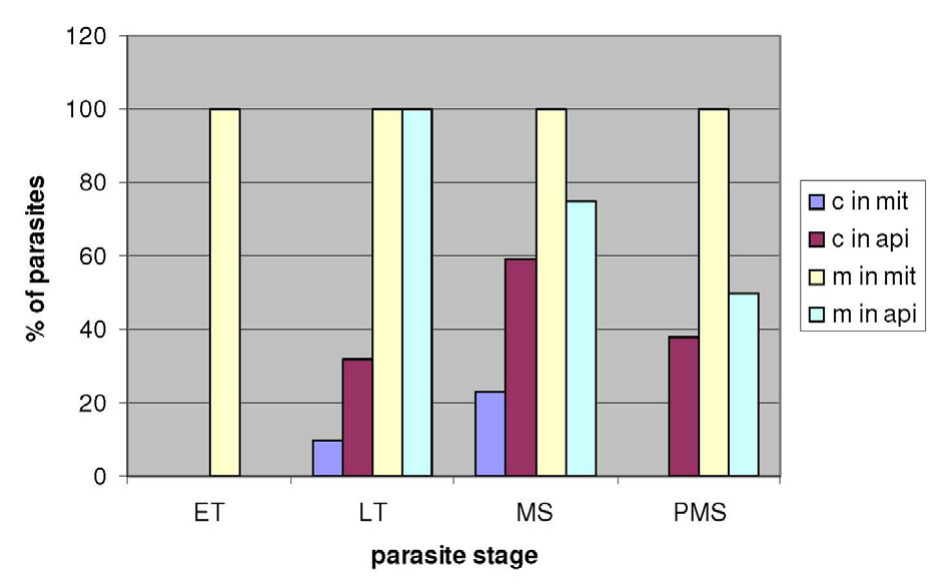
**Organellar distribution of fluorescence through the erythrocytic cycle**. Percentages of parasites (of the total number scanned for each stage) showing marked fluorescence for PfSHMTc (c) and PfSHMTm (m) in the mitochondrion (mit) and apicoplast (api). ET, early trophozoites; LT, late trophozoites; MS, mitotic schizonts; PMS, post-mitotic schizonts. For organellar localization of PfSHMTc, n = 82; for that of PfSHMTm, n = 76.

Use of anti-PfSHMTm in conjunction with anti-ACP showed that the PfSHMTm protein was also found within the apicoplast. The temporal distribution of PfSHMTm within the apicoplast through the erythrocytic cycle was qualitatively similar to that of PfSHMTc. Thus, there was no discernible co-localization seen in the early trophozoite (Figure 6A), however, there was a marked presence of PfSHMTm fluorescence within the apicoplasts of both late trophozoites and mitotic schizonts (Figure 6B and 6C, Figure 7; see also Figure 10). The later, post-mitotic, schizonts showed a similar lowering of apicoplast-associated PfSHMTm fluorescence to that found using the PfSHMTc specific antibody (Figure 6D). However, the spatial distribution of the PfSHMTm fluorescence within the elongating apicoplasts of early schizonts was, in contrast, dissimilar to that shown by PfSHMTc. Whereas the latter exhibited fluorescence relatively uniformly across the apicoplasts (Figure 4C and 4D; Figure 8B and 8C), PfSHMTm was distinctly concentrated in their extremities, and was notably absent, or in very much lower concentration, within the medial sections of these organelles (Figure 6C). This phenomenon is further illustrated by the sequential z plane views (at 0.2 μm intervals) through the same parasite shown in Figure 7B, especially in the second panel of this sequence, which clearly shows concentration of PfSHMTm fluorescence at the tips, and the fourth and fifth panels, where the lower degree of staining of the medial regions relative to the tips is apparent. The percentage co-localization of anti-PfSHMTm material with anti-ACP fluorescence was indicative of a low level of apicoplast PfSHMTm concentration in the trophozoite, e.g. 1.0% for Figure 6A, followed by much higher apicoplast PfSHMTm concentrations in the mitotically active schizont: e.g. 11.3% for Figure 6B, 11.8% for Figure 7A and 36.3% for Figure 6C. In the later, post-mitotic, schizonts, levels of co-localization fell back to lower values, the parasite shown in Figure 6D having a percentage of PfSHMTm material co-localizing with anti-ACP of only 0.3%.

### Imaging using an endogenously expressed apicoplast marker

The simultaneous use of two primary antibodies, even when raised in different species, combined with their respective fluorochrome-conjugated secondary antibodies, raised the formal possibility that any observed co-localization was the result of fortuitous interactions between those antibodies. To eliminate this possibility, 3D7 transfected parasites expressing the apicoplast-specific protein ACP fused to the DsRED reporter were employed [39]. When these parasites were probed with the single anti-PfSHMTc antibody, the images obtained showed an identical incidence of co-localization of the PfSHMTc fluorescence with the apicoplast (Figure 8A-C) as was seen using the two antibody approach above, although the relatively low absolute brightness of the DsRED fluorescence made these images unsuited to quantitative evaluation. The conclusion from this result is that the images created using two primary antibodies are a true reflection of the sub-cellular distribution of the proteins investigated and that the same distribution is found in two independent lines of the parasite, K1 and 3D7.

The parasites expressing PfACP-DsRED were also simultaneously probed with antibodies to both PfSHMTc (IgY) and PfSHMTm (IgG), again employing single plane scans without a DNA-specific dye rather than z-stacks for this triple labelling experiment. The parasite in Figure 5C (estimated to be a mid to late trophozoite), and that in Figure 5D (an early schizont) both show overlapping, though not identical, PfSHMTc and PfSHMTm fluorescence distribution in the cytoplasm. Both parasites show PfSHMTc and PfSHMTm coincident with the apicoplast as indicated by white colouration in the relevant merged image. Quantitative image analysis reinforces the visual indication of co-localization of both PfSHMTc and PfSHMTm with each other, and with the apicoplast specific fluorescence. In particular the apicoplast specific fluorescence was almost entirely (between 82.8% and 99.7%,) co-localized with the signals from both isoforms of SHMT.

### SHMT distribution in the post-mitotic schizont

In late, post-mitotic, schizonts, PfSHMTc fluorescence was characterized by a concentration in the central portion of the parasite. The peripheral regions of the parasite occupied by the nuclei and other constituents of the developing merozoites contained conspicuously lower levels of fluorescence, as shown in Figure 9A and 9B. The central area of late schizonts is the region that becomes the residual body upon completion of merozoite maturation and lysis of the erythrocyte, a prominent component of which is the pigment vacuole containing the crystalline haemozoin. In the very late schizont when the majority of the haemoglobin has been digested, the pigment vacuole occupies a large volume. Figure 9A and 9B show the central mass of dense haemozoin exhibiting no PfSHMTc staining but with marked PfSHMTc fluorescence in the region immediately surrounding it. To assess the relative frequency of this category of PfSHMTc distribution, 48 scans of post-mitotic schizonts were viewed, of which 15 showed a marked concentration of fluorescence in the centre of the schizont when compared to their periphery, an incidence of 31%. The use of anti-PfSHMTm antibody in conjunction with anti-PfSHMTc showed that PfSHMTm has a very similar concentration within the central region of the very late schizont (Figure 9C). Additional to this general distribution pattern, the post-mitotic schizont contains numerous small apicoplasts, each associated with a developing merozoite. Despite the diminutive size of these organelles, the persistence of PfSHMTc fluorescence within the 'daughter' apicoplasts was still discernible in some images, e.g. in Figure 9B, where the lower right plastid in the parasite clearly shows its presence. The three-dimensional projection shown in Figure 9B is interesting as it shows a relatively late stage of daughter merozoite biogenesis.

### Nuclear localization

In most parasites viewed there was a distinctly lower PfSHMTc fluorescence within nuclei than was found in the cytoplasm. However, PfSHMTc fluorescence was very rarely entirely excluded from the nucleus (see especially Figure 4C and 4D; Figure 9B and 9C). The level of nuclear relative to cytoplasmic fluorescence was variable with higher levels of intranuclear PfSHMTc fluorescence seen in some late trophozoites and mitotic schizonts. Nuclear PfSHMTc fluorescence rarely approached the intensity of cytoplasmic fluorescence, however. In contrast, PfSHMTm showed very little evidence of nuclear localization throughout the erythrocytic cycle, with most images showing an essentially complete exclusion of PfSHMTm fluorescence from nuclei (Figure 3D and 3E; Figure 6A, C and 6D).

### Relative incidence of organellar SHMT fluorescence through the erythrocytic cycle

In view of the initially surprising results that PfSHMTc showed organellar co-localization patterns, a large number of z-axis scans of parasites were analysed in order to ascertain the relative incidence of organellar fluorescence for this isoform over the erythrocytic cycle. Parasites were assigned to one of four broadly defined developmental stages by examination of overall size, haemozoin development and nuclear morphology (Figure 10). These results emphasize the stage-specific dependence of organellar PfSHMTc fluorescence, which was undetectable in parasites up to and including the early trophozoite stages, visible from mid-trophozoites onward and peaking at the mitotic schizont stages. The corresponding analysis for PfSHMTm with respect to the mitochondrion is strikingly different in that 100% of parasites showed fluorescence in this organelle, regardless of the cell cycle stage. However, its incidence in the apicoplast was similar to that of PfSHMTc, in that it was not seen in the early trophozoite stage but peaked in the late trophozoite stage, although the percentage of parasites displaying this pattern was significantly higher than was the case for PfSHMTc.

### GFP-tagging of SHMT via transfection

**Figure 11:**
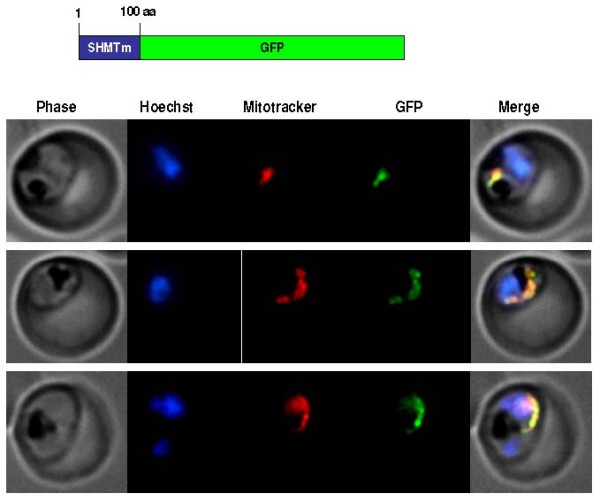
**GFP-tagging of truncated PfSHMTm in transfected 3D7 parasites**. Fluorescence images of parasites transfected to yield a GFP-fusion carrying the first 100 amino acids of PfSHMTm at the N-terminus. MitoTracker was also used to localize the mitochondrion, which showed complete coincidence with the GFP fluorescence (three examples shown).

To support the immunofluorescence studies in a complementary manner, independent attempts were made in the two collaborating laboratories to produce transfected parasites expressing GFP-tagged, full length PfSHMTc and PfSHMTm endogenously, as well as shorter versions carrying a GFP-tag downstream of the first 100 amino acids of each protein (i.e. about one-quarter of their total length). Despite repeated transfections using several different protocols for these four constructs, viable parasites could only ever be recovered in the case of the truncated version of PfSHMTm + GFP. Fluorescence microscopy clearly located this hybrid protein in the mitochondrion (Figure 11), confirming the initial prediction based on sequence analysis that PfSHMTm carries a mitochondrial targeting signal at its N-terminus [24]. However, in contrast to the studies above using the anti-PfSHMTm antibody, no additional distribution in the cytoplasm or apicoplast was apparent, suggesting that localization to these areas was dependent upon properties of the full-length molecule.

### Transcript analysis of PfSHMTc

In the original characterization of the gene encoding PfSHMTc, comparison of cDNA and genomic sequences, together with RACE analyses of the transcript start point in two independent laboratories [8,21], identified only a ~240 base 5' UTR on the mRNA which lacked any AUG motif upstream of the documented start codon. To confirm and extend this result, we carried out RT-PCR experiments using a range of internal primers based on genomic sequence extending up to 1 kb upstream of the start codon. However, no splice variants were detected (data not shown), nor could any putative splicing event using the normal GU and AG intron junction signals within this sequence create an alternative start codon. Thus there was no evidence that PfSHMTc might employ a conventional signal sequence that had previously been overlooked to gain access to the organellar compartments.

## Discussion

SHMT is the principal agent by which one-carbon units are introduced onto folate carriers for subsequent essential transfer reactions, including the formation of thymidylate. Of the two SHMT isoforms expressed by *P. falciparum*, PfSHMTc is an enzymatically active member of the thymidylate cycle [21-23], whereas PfSHMTm is more enigmatic, as it lacks most of the conserved active site residues found in other SHMTs, whether cytoplasmic or organellar [24,42] and has been found to be inactive in studies of the recombinant protein [23]. With a combination of antibody probes and endogenous expression of tagged molecules, the cellular distribution of these two species across the parasite erythrocytic cycle has been investigated to gain possible insight into their biological function.

Although PfSHMTc lacks any obvious signal sequences and was expected to be confined to the cytoplasm, the most surprising result was that, although it is indeed in the cytoplasm that the majority population is found throughout erythrocytic development, PfSHMTc also localizes within parasite organelles, including the mitochondrion and particularly the apicoplast. Moreover, the distribution varies in a dynamic and developmental stage-dependent manner, consistent with a temporally mediated variation in the targeting of newly expressed enzyme protein between sub-cellular locations from a cytoplasmic pool. PfSHMTm, on the other hand, was identified as having an N-terminal mitochondrial targeting sequence [24], which was functionally confirmed here using a transfected parasite construct expressing GFP fused to its N-terminal domain. However, full-length PfSHMTm in its native state shows complex localization patterns that are similar to, but subtly different from those of PfSHMTc in the cytoplasm, mitochondrion and apicoplast.

Although the parasite preparations undergo a multi-step protocol for immunofluorescence that necessarily includes a mild detergent permeabilization, there are strong reasons to believe that the results obtained are not artifactual. Thus (i) organellar fine structures of the parasites are preserved, (ii) both primary anti-PfSHMT antibodies show a high degree of specificity, (iii) antibodies against two known cytoplasmically located enzymes show no organellar co-localization, (iv) conversely, the anti-ACP primary antibody locates exclusively to the apicoplast as expected, with no evidence of staining elsewhere that might indicate a loss of organellar integrity, (v) the incidence of PfSHMTc in the organelles shows a strong stage-dependency, being absent in early trophozoites, peaking in late trophozoites and mitotic schizonts, then diminishing in later (post-mitotic) schizonts, (vi) a different stage dependency for PfSHMTm is evident, particularly in the case of the mitochondrion, where co-localization is seen in all parasites throughout the cycle, and (vii) localization patterns of PfSHMTc within the apicoplast are the same, regardless of whether two primary antibodies (anti-PfSHMTc and anti-PfACP) are employed as probes or anti-PfSHMTc alone plus the endogenous apicoplast fluorescence from transfected parasites in which PfACP is tagged with DsRED. The use of z-stack scanning and quantitation of overlapping fluorescence signals considerably increased confidence in assigning position compared to conventional 2 D analysis of images in a single plane, especially in the case of late trophozoites and early schizonts, where organelles were large enough to permit at least 3 and up to 7 or 8 planes within the organelle to be examined, as exemplified in Figure 7. Stages at which a specifically stained organelle was at its smallest unavoidably gave less clear-cut images that could be more easily compromised by adjacent cytoplasmic staining. Moreover the close apposition of the mitochondrion and apicoplast is also potentially problematic when both are very small or narrow, although this was less of an issue with the larger organelles of K1, primarily used for this study, compared to 3D7. However, attempts to increase resolution further by scanning z-planes every 0.1 μm instead of 0.2 μm resulted in unacceptable levels of photobleaching before such lengthy scans could be completed.

The observed association of PfSHMTc with the mitochondrion and the apicoplast is not of equal degree. The mitochondrion shows relatively low levels of PfSHMTc fluorescence in the earlier and later stages of the cycle, with signal within the organellar lumen only obvious in the late trophozoite and early schizont stages, when the mitochondrion is expanding and then elongating, with significant synthesis of internal constituents [45]. However, even at maximum visibility, the fluorescence intensity, as confirmed by quantitative image analysis, does not exceed that found in the surrounding cytoplasm. In contrast to the mitochondrion, the apicoplast shows a significantly higher level of PfSHMTc association over a longer period of the erythrocytic cycle. PfSHMTc fluorescence within the apicoplast lumen is first detected in trophozoite stages before the organelle has expanded noticeably and persists into the small daughter plastids of very late schizonts. In both of these developmental stages, the concentration of PfSHMTc within the apicoplast does not exceed that in the cytoplasm. However, between these stages, in the early schizont, when the apicoplast is expanding maximally and subsequently elongating, PfSHMTc fluorescence inside this organelle becomes very marked, such that the concentration of PfSHMTc within it now exceeds that in the surrounding cytoplasm. The percentage of total PfSHMTc fluorescence co-localising with apicoplast-specific fluorescence was measured in some parasites at this stage at >20%, a considerable proportion of the cellular total.

The PfSHMTm protein, despite its N-terminal mitochondrial targeting sequence, shows a similar spatial and temporal distribution to that of PfSHMTc, albeit with some important variation. Thus, the level of localization of PfSHMTm within the mitochondrion is distinctly higher than that of PfSHMTc. Moreover, the occurrence of concentrations of PfSHMTm within the extremities of elongating apicoplasts and a corresponding paucity in the medial sections is a polarization not seen with Pf SHMTc and may suggest a more specific developmental role for PfSHMTm in this organelle. Given also the occurrence of Pf SHMTm in the cytoplasm, there are likely to be further signals downstream of the N-terminal domain yet to be characterized that contribute to the complex and dynamic distribution patterns, as discussed further below.

The data suggest a connection between these apparently rather short-lived associations and internal organellar metabolism. Both organelles must replicate their own genomes, prior to which local demand for folate pathway products would be high. Moreover, there is evidence for the existence of a glycine cleavage complex (GCV) in the mitochondrion [24], which is dependent on the provision of folate cofactor. Studies in other systems indicate that substituted tetrahydrofolates cannot freely exchange between mitochondrial and cytoplasmic compartments [14], suggesting that transport of folate enzymes into membrane-bound organelles may be essential. In the very late post-mitotic schizont, PfSHMTc is found concentrated in the centre of the parasite, in the region that forms the residual body on erythrocyte lysis and merozoite release. This association can be rationalized in that, after mitosis, demand for DNA precursors is low, however, the late schizont is very active in protein production for organellogenesis and other aspects of merozoite maturation. As amino acids are released from haemoglobin, the late concentration of PfSHMTc in and around this organelle may be connected with an increased demand for the reversible Ser/Gly interconversion function of the SHMT enzyme and/or methionine metabolism.

Although plant cells also exhibit a partitioning of SHMT across cytoplasm, mitochondrion and plastid, this association is a persistent feature [46], rather than the more transient phenomenon seen here in the malaria parasite. By contrast, the parasite must undergo rapid asexual reproduction at the blood stages, requiring parasite metabolism to be highly efficient in its production and use of folate pathway components. The locations of greatest demand for such products would thus vary through the processes of growth, repeated mitoses and cytodifferentiation in the erythrocytic cycle. It would, therefore, be advantageous to be able to translocate folate enzymes to varying subcellular locations as the demand for folates changed throughout development. The results of this study support a view of the cellular location of folate pathway enzymes being dynamic and responsive to the changing needs of the parasite over time.

**Figure 12:**
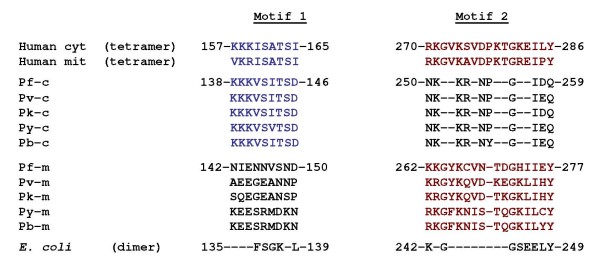
**Potential interactions between PfSHMTc and PfSHMTm**. The two protein sequence motifs identified in mammalian SHMTs (both cytoplasmic and mitochondrial isoforms; top two lines) essential for stable tetramer formation; bacterial SHMTs lack these motifs entirely and form stable dimers (bottom line; *E. coli *as example) [42]. SHMTc from *P. falciparum *and its orthologues from other *Plasmodium *species (*P. vivax*, *P. knowlesi*, *P. yoelii *and *P. berghei*, respectively) possess a highly conserved equivalent of Motif 1 only (blue text), whereas the SHMTm forms possess only Motif 2 (red text), suggesting that heterotetramers could be formed by a combination of (PfSHMTc)_2 _and (PfSHMTm)_2_.

Important questions that now need to be addressed are how PfSHMTm and, more puzzling, PfSHMTc, are targeted to the organelles investigated here and their precise function(s) therein. Dual targeting of proteins to the mitochondrion and plastid is frequently observed in other systems [47]. However, in organisms as diverse as plants, yeast and mammals, dedicated cytoplasmic and organellar isoforms of SHMT are employed to effect compartmentalized folate metabolism. Only PfSHMTm has a recognizable mitochondrial targeting sequence and neither isoform possesses the conventional bipartite topogenic signal associated with plastid targeting via the endoplasmic reticulum in *P. falciparum *and the closely related *Toxoplasma gondii *[48,49]. Moreover, although a proportion of PfSHMTm is located in the mitochondrion throughout the erythrocytic cycle and also in the apicoplast in the middle to late stages, how can this isoform perform the necessary enzymic steps when its sequence (see Additional file 2 Sequence alignments of the PfSHMT isoforms) and the inactivity of the recombinant protein [23] strongly indicate that it cannot? It could be that the folate metabolism essential for the replication of their genomes is provided by the enzymatically competent PfSHMTc after gaining access to these organelles. The sequence and transcript analyses exclude the possibility of a hitherto unidentified upstream leader sequence that could splice onto the ORF as currently defined, but cryptic motifs further downstream in the encoded protein cannot be excluded, especially as numerous plastid proteins in other systems depend upon ill-defined, sometimes non-contiguous regions of the molecule [50]. However, another intriguing possibility is suggested by a closer analysis of the primary sequences of PfSHMTc and PfSHMTm, which reveals that each carries one or the other of two internal conserved sequence motifs (residues 138-146 in PfSHMTc and residues 262-277 in PfSHMTm), both of which are present in the SHMTs of higher organisms and are known to mediate intersubunit interactions (Figure 12). Thus, mammalian SHMTs are stable homotetramers, whereas bacterial SHMTs, which lack these motifs entirely, are homodimers [42]. The plasmodial proteins seem to represent a complementary pairing system thus far unique among SHMT types and this leads us to the hypothesis that while PfSHMTm is in itself apparently catalytically inactive, a dimer thereof might be able to form a stable but readily reversible heterotetramer with dimeric PfSHMTc, generating a complex in which the requisite SHMT activity is provided (from PfSHMTc) together with (an) organellar targeting sequence(s) (from PfSHMTm) that could be modulated as necessary. Such an arrangement would have parallels with the S-adenosyl-L-methionine decarboxylase (AdoMetDC) system of trypanosomes, where a catalytically inactive paralogue of AdoMetDC forms a heterodimer with AdoMetDC itself and thereby regulates the activity of the latter allosterically [51]. This scenario could explain why the localizations of the two PfSHMT isoforms show considerable overlap, and why our GFP construct attached to the first 100 amino acids of PfSHMTm, and thus lacking both of the above motifs, migrates solely to the mitochondrion. Moreover, many nuclear-encoded proteins destined for plastids and mitochondria in other organisms are translated on cytoplasmic ribosomes and imported (often long) after their synthesis [47], thus providing a credible precedent for encounters between PfSHMTc and PfSHMTm. However, it is certainly unclear at this point precisely how such a complex could be successfully translocated across the requisite membranes, although analyses of organellar protein transport found in other systems serve to emphasize the considerable diversity of mechanisms associated with this phenomenon [50,52].

## Conclusions

The two isoforms of SHMT in *P. falciparum*, PfSHMTc and PfSHMTm, exhibit complex distribution patterns across the cytoplasm and organelles of the parasite that are similar but differ in their levels of occupancy and cell-cycle stage dependency. PfSHMTm is confirmed as having an N-terminal mitochondrial targeting sequence whereas PfSHMTc lacks any obvious targeting signals. Interactions between the two isoforms suggested by sequence analysis may be involved in the dynamic patterns of localization observed and may be important in overcoming the apparent lack of catalytic competence of the PfSHMTm isoform. Further studies are required to establish whether such an association can occur and play a role in ensuring the provision of essential folate cofactors for replication of the nuclear, mitochondrial and apicoplast genomes.

## Competing interests

The authors declare that they have no competing interests.

## Authors' contributions

MR developed the immunofluorescence protocols, carried out most of the experimental work in Manchester and drafted the manuscript. IBM constructed GFP transfection plasmids, produced parasite transfectants and carried out the imaging thereof. SLM contributed to the establishment of the immunofluorescence procedures. PFGS and JEH conceived the study, wrote parts of the manuscript and prepared the final version. All authors were involved in data interpretation and analysis and have approved the final manuscript.

## Supplementary Material

Additional file 1 **Negative control images for organellar staining**. The figure shows immunofluorescence images obtained using antibodies against known cytoplasmic enzymes. Click here for file

Additional file 2 **Sequence alignments of the PfSHMT isoforms**. The figure shows alignment of the PfSHMTc and PfSHMTm sequences with cytoplasmic and mitochondrial SHMTs from other organisms. Click here for file

## References

[B1] Snow RW Guerra CA Noor AM Myint HY Hay SI The global distribution of clinical episodes of *Plasmodium falciparum *malaria Nature 2005 434 214 217 10.1038/nature03342 15759000 PMC3128492

[B2] Hyde JE Drug-resistant malaria - an insight FEBS Journal 2007 274 4688 4698 10.1111/j.1742-4658.2007.05999.x 17824955 PMC2720519

[B3] Müller IB Hyde JE Antimalarial drugs: modes of action and mechanisms of parasite resistance Future Microbiology 2010 5 1857 1875 10.2217/fmb.10.136 21155666

[B4] Hyde JE Exploring the folate pathway in *Plasmodium falciparum* Acta Trop 2005 94 191 206 15845349 10.1016/j.actatropica.2005.04.002 PMC2720607

[B5] Krungkrai J Webster HK Yuthavong Y De novo and salvage biosynthesis of pteroylpentaglutamates in the human malaria parasite, *Plasmodium falciparum* Mol Biochem Parasitol 1989 32 25 37 10.1016/0166-6851(89)90126-6 2643036

[B6] Wang P Nirmalan N Wang Q Sims PFG Hyde JE Genetic and metabolic analysis of folate salvage in the human malaria parasite *Plasmodium falciparum* Mol Biochem Parasitol 2004 135 77 87 10.1016/j.molbiopara.2004.01.008 15287589

[B7] Wang P Read M Sims PFG Hyde JE Sulfadoxine resistance in the human malaria parasite *Plasmodium falciparum *is determined by mutations in dihydropteroate synthetase and an additional factor associated with folate utilization Mol Microbiol 1997 23 979 986 10.1046/j.1365-2958.1997.2821646.x 9076734

[B8] Lee CS Salcedo E Wang Q Wang P Sims PFG Hyde JE Characterization of three genes encoding enzymes of the folate biosynthetic pathway in *Plasmodium falciparum* Parasitology 2001 122 1 13 10.1017/S0031182000006946 11197757

[B9] Salcedo E Cortese JF Plowe CV Sims PFG Hyde JE A bifunctional dihydrofolate synthetase-folylpolyglutamate synthetase in *Plasmodium falciparum *identified by functional complementation in yeast and bacteria Mol Biochem Parasitol 2001 112 241 254 10.1016/S0166-6851(00)00370-4 11223131

[B10] Wang P Wang Q Yang Y Coward JK Nzila A Sims PFG Hyde JE Characterisation of the bifunctional dihydrofolate synthase-folylpolyglutamate synthase from *Plasmodium falciparum*; a potential novel target for antimalarial antifolate inhibition Mol Biochem Parasitol 2010 172 41 51 10.1016/j.molbiopara.2010.03.012 20350571 PMC2877875

[B11] Krumdieck CL Eto I Baggott JE Regulatory role of oxidized and reduced pteroylpolyglutamates Ann NY Acad Sci 1992 669 44 58 10.1111/j.1749-6632.1992.tb17088.x 1444059

[B12] Hanson AD Gregory JF Synthesis and turnover of folates in plants Curr Opin Plant Biol 2002 5 244 249 10.1016/S1369-5266(02)00249-2 11960743

[B13] Rebeille F Ravanel S Jabrin S Douce R Storozhenko S Van Der Straeten D Folates in plants: biosynthesis, distribution, and enhancement Physiologia Plantarum 2006 126 330 342 10.1111/j.1399-3054.2006.00587.x

[B14] Schirch V Strong WB Interaction of folylpolyglutamates with enzymes in one-carbon metabolism Arch Biochem Biophys 1989 269 371 380 10.1016/0003-9861(89)90120-3 2645826

[B15] Chen LF Chan SY Cossins EA Distribution of folate derivatives and enzymes for synthesis of 10-formyltetrahydrofolate in cytosolic and mitochondrial fractions of pea leaves Plant Physiology 1997 115 299 309 10.1104/pp.115.3.1127 12223808 PMC158486

[B16] Christensen KE MacKenzie RE Mitochondrial one-carbon metabolism is adapted to the specific needs of yeast, plants and mammals Bioessays 2006 28 595 605 10.1002/bies.20420 16700064

[B17] Gagnon D Foucher A Girard I Ouellette M Stage specific gene expression and cellular localization of two isoforms of the serine hydroxymethyltransferase in the protozoan parasite *Leishmania* Mol Biochem Parasitol 2006 150 63 71 10.1016/j.molbiopara.2006.06.009 16876889

[B18] Girgis S Nasrallah IM Suh JR Oppenheim E Zanetti KA Mastri MG Stover PJ Molecular closing, characterization and alternative splicing of the human cytoplasmic serine hydroxymethyltransferase gene Gene 1998 210 315 324 10.1016/S0378-1119(98)00085-7 9573390

[B19] Tendler SJB Threadgill MD Tisdale MJ Activities of serine hydroxymethyltransferase in murine tissues and tumors Cancer Letters 1987 36 65 69 10.1016/0304-3835(87)90103-0 3581057

[B20] Douce R Bourguignon J Neuburger M Rebeille F The glycine decarboxylase system: a fascinating complex Trends Plant Sci 2001 6 167 176 10.1016/S1360-1385(01)01892-1 11286922

[B21] Alfadhli S Rathod PK Gene organization of a *Plasmodium falciparum *serine hydroxymethyltransferase and its functional expression in *Escherichia coli* Mol Biochem Parasitol 2000 110 283 291 10.1016/S0166-6851(00)00282-6 11071283

[B22] Maenpuen S Sopitthummakhun K Yuthavong Y Chaiyen P Leartsakulpanich U Characterization of *Plasmodium falciparum *serine hydroxymethyltransferase - a potential antimalarial target Mol Biochem Parasitol 2009 168 63 73 10.1016/j.molbiopara.2009.06.010 19591881

[B23] Pang CKT Hunter JH Gujjar R Podutoori R Bowman J Mudeppa DG Rathod PK Catalytic and ligand-binding characteristics of *Plasmodium falciparum *serine hydroxymethyltransferase Mol Biochem Parasitol 2009 168 74 83 10.1016/j.molbiopara.2009.06.011 19591883 PMC2741015

[B24] Salcedo E Sims PFG Hyde JE A glycine-cleavage complex as part of the folate one-carbon metabolism of *Plasmodium falciparum* Trends Parasitol 2005 21 406 411 10.1016/j.pt.2005.07.001 16039160 PMC2719866

[B25] Spalding MD Allarya M Gallaghera JR Prigge ST Validation of a modified method for Bxb1 mycobacteriophage integrase-mediated recombination in *Plasmodium falciparum *by localization of the H-protein of the glycine cleavage complex to the mitochondrion Mol Biochem Parasitol 2010 172 156 160 10.1016/j.molbiopara.2010.04.005 20403390 PMC2875341

[B26] Read M Sherwin T Holloway SP Gull K Hyde JE Microtubular organization visualized by immunofluorescence microscopy during erythrocytic schizogony in *Plasmodium falciparum *and investigation of post-translational modifications of parasite tubulin Parasitology 1993 106 223 232 10.1017/S0031182000075041 8488059

[B27] Margos G Bannister LH Dluzewski AR Hopkins J Williams IT Mitchell GH Correlation of structural development and differential expression of invasion-related molecules in schizonts of *Plasmodium falciparum* Parasitology 2004 129 273 287 10.1017/S0031182004005657 15471003

[B28] Doerig C Chakrabarti D Waters AP, Janse CJ Cell cycle control in *Plasmodium falciparum*: a genomics perspective Malaria Parasites: Genomes and Molecular Biology 2004 Wymondham, UK: Caister Academic Press 249 287

[B29] Nirmalan N Wang P Sims PFG Hyde JE Transcriptional analysis of genes encoding enzymes of the folate pathway in the human malaria parasite *Plasmodium falciparum* Mol Microbiol 2002 46 179 190 10.1046/j.1365-2958.2002.03148.x 12366841

[B30] Nirmalan N Flett F Skinner T Hyde JE Sims PFG Microscale solution isoelectric focusing as an effective strategy enabling containment of hemeoglobin-derived products for high-resolution gel-based analysis of the *Plasmodium falciparum *proteome J Proteome Res 2007 6 3780 3787 10.1021/pr070278r 17696383 PMC2632839

[B31] O'Cualain RDM Hyde JE Sims PFG A protein-centric approach for the identification of folate enzymes from the malarial parasite, *Plasmodium falciparum*, using OFFGEL™ solution-based isoelectric focussing and mass spectrometry Malar J 2010 9 286 20955557 10.1186/1475-2875-9-286 PMC2967559

[B32] Sherwin T Read M Hyde JE Immunofluorescence of parasites Protocols in Molecular Parasitology 1993 21 Totowa, New Jersey: Humana Press 407 414 *Methods in Molecular Biology*full_text

[B33] Read M Hyde JE Hyde JE Simple *in vitro *cultivation of the malaria parasite *Plasmodium falciparum *(erythrocytic stages) suitable for large-scale preparations Protocols in Molecular Parasitology 1993 21 Totowa, New Jersey: Humana Press 43 55 *Methods in Molecular Biology*full_text 10.1385/0-89603-239-6:43 8220733

[B34] Naughton JA Bell A Studies on cell-cycle synchronization in the asexual erythrocytic stages of *Plasmodium falciparum* Parasitology 2007 134 331 337 10.1017/S0031182006001466 17034650

[B35] Müller IB Knöckel J Eschbach ML Bergmann B Walter RD Wrenger C Secretion of an acid phosphatase provides a possible mechanism to acquire host nutrients by *Plasmodium falciparum* Cell Microbiol 2010 12 677 691 20070315 10.1111/j.1462-5822.2010.01426.x

[B36] Crabb BS Rug M Gilberger T-W Thompson JK Triglia T Maier AG Cowman AF Transfection of the human malaria parasite *Plasmodium falciparum* Methods Mol Biol 2004 270 263 276 15153633 10.1385/1-59259-793-9:263

[B37] Waller RF Keeling PJ Donald RGK Striepen B Handman E Lang-Unnasch N Cowman AF Besra GS Roos DS McFadden GI Nuclear-encoded proteins target to the plastid in *Toxoplasma gondii *and *Plasmodium falciparum* Proc Natl Acad Sci USA 1998 95 12352 12357 10.1073/pnas.95.21.12352 9770490 PMC22835

[B38] Fitzpatrick T Ricken S Lanzer M Amrhein N Macheroux P Kappes B Subcellular localization and characterization of chorismate synthase in the apicomplexan *Plasmodium falciparum* Mol Microbiol 2001 40 65 75 10.1046/j.1365-2958.2001.02366.x 11298276

[B39] Sato S Wilson R The use of DsRED in single- and dual-color fluorescence labeling of mitochondrial and plastid organelles in *Plasmodium falciparum* Mol Biochem Parasitol 2004 134 175 179 10.1016/j.molbiopara.2003.11.015 14747157

[B40] Costes SV Daelemans D Cho EH Dobbin Z Pavlakis G Lockett S Automatic and quantitative measurement of protein-protein colocalization in live cells Biophysical Journal 2004 86 3993 4003 10.1529/biophysj.103.038422 15189895 PMC1304300

[B41] Lee SW Lee HW Chung HJ Kim YA Kim YJ Hahn Y Chung JH Park YS Identification of the genes encoding enzymes involved in the early biosynthetic pathway of pteridines in *Synechocystis *sp PCC 6803 FEMS Microbiol Lett 1999 176 169 176 10.1111/j.1574-6968.1999.tb13658.x 10418143

[B42] Franca TCC Pascutti PG Ramalho TC Figueroa-Villar JD A three-dimensional structure of *Plasmodium falciparum *serine hydroxymethyltransferase in complex with glycine and 5-formyl- tetrahydrofolate. Homology modeling and molecular dynamics Biophys Chem 2005 115 1 10 10.1016/j.bpc.2004.12.002 15848278

[B43] Hopkins J Fowler R Krishna S Wilson I Mitchell G Bannister L The plastid in *Plasmodium falciparum *asexual blood stages: a three-dimensional ultrastructural analysis Protist 1999 150 283 295 10.1016/S1434-4610(99)70030-1 10575701

[B44] Kobayashi T Sato S Takamiya S Komaki-Yasuda K Yano K Hirata A Onitsuka I Hata M Mi-Ichi F Tanaka T Mitochondria and apicoplast of *Plasmodium falciparum*: Behaviour on subcellular fractionation and the implication Mitochondrion 2007 7 125 132 10.1016/j.mito.2006.11.021 17289446

[B45] van Dooren GG Marti M Tonkin CJ Stimmler LM Cowman AF McFadden GI Development of the endoplasmic reticulum, mitochondrion and apicoplast during the asexual life cycle of *Plasmodium falciparum* Mol Microbiol 2005 57 405 419 10.1111/j.1365-2958.2005.04699.x 15978074

[B46] Besson V Neuburger M Rebeille F Douce R Evidence for three serine hydroxymethyltransferases in green leaf cells - purification and characterization of the mitochondrial and chloroplastic isoforms Plant Physiology and Biochemistry 1995 33 665 673

[B47] Peeters N Small I Dual targeting to mitochondria and chloroplasts Biochimica Et Biophysica Acta-Molecular Cell Research 2001 1541 54 63 10.1016/S0167-4889(01)00146-X 11750662

[B48] Foth BJ Ralph SA Tonkin CJ Struck NS Fraunholz M Roos DS Cowman AF McFadden GI Dissecting apicoplast targeting in the malaria parasite Plasmodium falciparum Science 2003 299 705 708 10.1126/science.1078599 12560551

[B49] Tonkin CJ Kalanon M McFadden GI Protein targeting to the malaria parasite plastid Traffic 2008 9 166 175 17900270 10.1111/j.1600-0854.2007.00660.x

[B50] Inaba T Schnell DJ Protein trafficking to plastids: one theme, many variations Biochem J 2008 413 15 28 10.1042/BJ20080490 18537794

[B51] Willert EK Fitzpatrick R Phillips MA Allosteric regulation of an essential trypanosome polyamine biosynthetic enzyme by a catalytically dead homolog Proc Natl Acad Sci USA 2007 104 8275 8280 10.1073/pnas.0701111104 17485680 PMC1895940

[B52] Bolte K Bullmann L Hempel F Bozarth A Zauner S Maier UG Protein targeting into secondary plastids J Eukaryot Microbiol 2009 56 9 15 10.1111/j.1550-7408.2008.00370.x 19335770

