# Supplementary material for: Dynamic subcellular localization of isoforms of the folate pathway enzyme serine hydroxymethyltransferase (SHMT) through the erythrocytic cycle of Plasmodium falciparum
Source: Malar J. 2010 Dec 3;9:351. doi: 10.1186/1475-2875-9-351 (PMC3014972; doi:10.1186/1475-2875-9-351)
Supplement: Additional file 1 — Negative control images for organellar staining. The figure shows immunofluorescence images obtained using antibodies against known cytoplasmic enzymes. [file 1475-2875-9-351-S1.PDF]

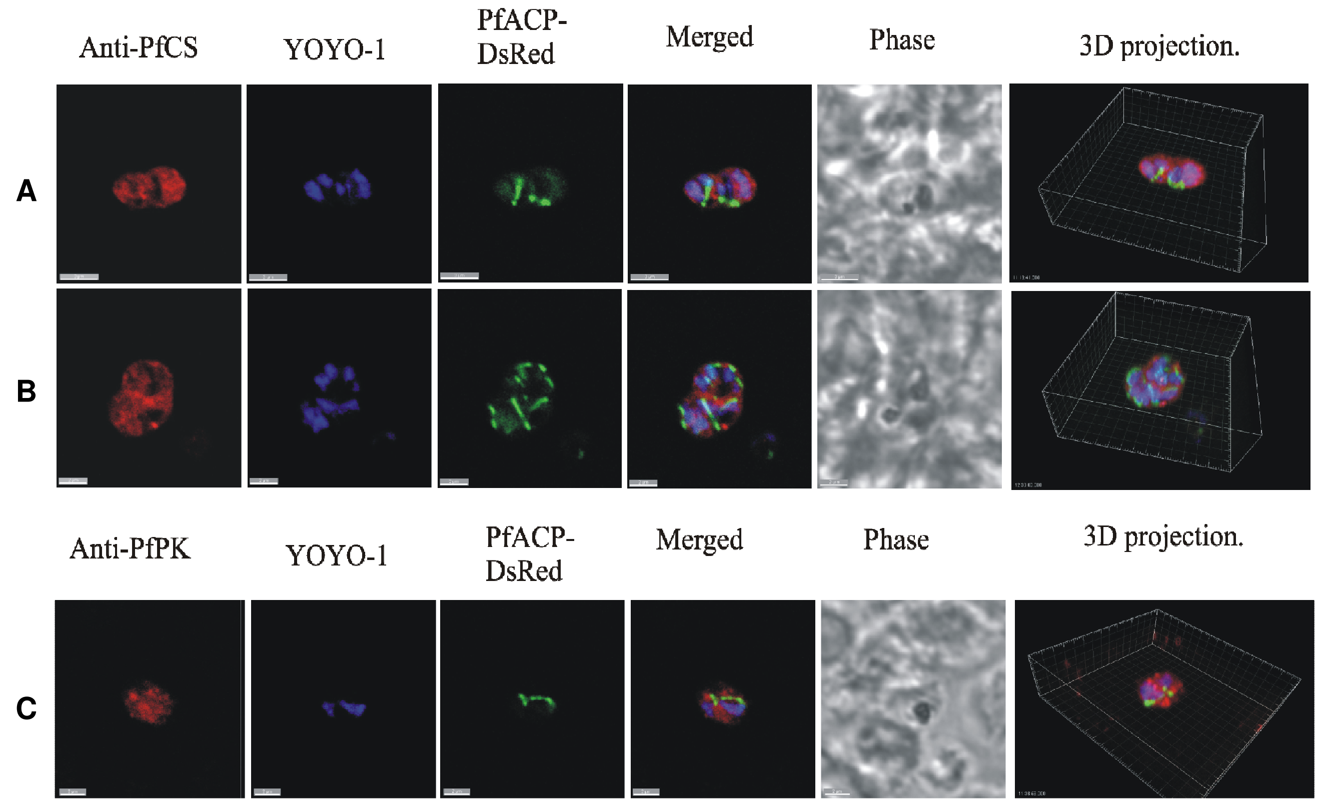

Supplementary Figure 1 - Negative control images for organellar staining.

A and **B.** anti-PfCS (chorismate synthase), **C.** anti-PfPK (cyclin-dependent protein kinase 5), both enzymes previously characterised as having a simple cytoplasmic distribution {Fitzpatrick, 2001 #618}. All parasites were expressing DsRED labelled PfACP. The control antibodies show little or no fluorescence coincident with the apicoplasts, despite the parasites being in the mitotic schizont phase where anti-PfSHMT fluorescence is most overt (scale bars **A** 3 μm, **B** and **C** 2 μm).
