# Supplementary material for: Dynamic subcellular localization of isoforms of the folate pathway enzyme serine hydroxymethyltransferase (SHMT) through the erythrocytic cycle of Plasmodium falciparum
Source: Malar J. 2010 Dec 3;9:351. doi: 10.1186/1475-2875-9-351 (PMC3014972; doi:10.1186/1475-2875-9-351)
Supplement: Additional file 2 — Sequence alignments of the PfSHMT isoforms. The figure shows alignment of the PfSHMTc and PfSHMTm sequences with cytoplasmic and mitochondrial SHMTs from other organisms. [file 1475-2875-9-351-S2.PDF]

```
                 *        20         *        40         *
arabidC : -----MALALRRLSSSVKK---PISLLSS-------------NGGSLRFMWIKQLNA :  36
peaM    : ---MAMAMALRKLSSSVNKSSRPLFSASSLYYKSSLPDEAVYDKENPRVTWPKQLNS :  54
humanC  : -------------------MTMPVNGAHK--------DADLWSSHDK---MLAQP-- :  25
humanM  : MLYFSLFWAARPLQRCGQLVRMAIRAQHS--------NAAQTQTGEANRGWTGQES- :  48
yeastC  : -------------------------------------------MPYTLSDAHHKLITS :  15
yeastM  : -----------------------MFPRASALAK---CMATVHRRGLLTSGAQSLVSK :  31
PfSHMTc : ---------------------------------------------------MFNNDP-- :   6
PfSHTMm : ----------------------------------------------MLKEFVKNVNVR :  12


       60         *        80         *       100          *
arabidC : SLDEIDPEVADIIE---LEKARQWKGFELIPSENFTSLSVMQAVGSVMTNKYSEGYP :  90
peaM    : PLEVIDPEIADIIE---LEKARQWKGLELIPSENFTSLSVMQAVGSVMTNKYSEGYP : 108
humanC  : -LKDSDVEVYNIIK---KESNRQRVGLELIASENFASRAVLEALGSCLNNKYSEGYP :  78
humanM  : -LSDSDPEMWELLQ---REKDRQCRGLELIASENFCSRAALEALGSCLNNKYSEGYP : 101
yeastC  : HLVDTDPEVDSIIK---DEIERQKHSIDLIASENFTSTSVFDALGTPLSNKYSEGYP :  69
yeastM  : PVSEGDPEMFDILQ---QERHRQKHSITLIPSENFTSKAVMDLLGSELQNKYSEGYP :  85
PfSHMTc : -LQKYDKELFDLLE---KEKNRQIETINLIASENLTNTAVRECLGDRISNKYSEGYP :  59
PfSHTMm : N-HRYIS--FYSLRNQSRLNDIDDETYNMLKSYKNKND-INLSLVHNIMPTYMKEYL :  65
                                 &                          & #

          120        *       140         *       160          *
arabidC : GARYYGGNEYVVCILLTRYIDMAETLCQKRALEAFQLDPSKWGVNVQSLSGSPANFQ : 147
peaM    : GARYYGGNE---------YIDMAETLCQKRALEAFRLDPAKWGVNVQPLSGSPSNFQ : 156
humanC  : GQRYYGGTE---------FIDELETLCQKRALQAYKLDPQCWGVNVQPYSGSPANFA : 126
humanM  : GKRYYGGAE---------VVDEIELLCQRRALEAFDLDPAQWGVNVQPYSGSPANLA : 149
yeastC  : GARYYGGNE---------HIDRMEILCQQRALKAFHVTPDKWGVNVQTLSGSPANLQ : 117
yeastM  : GERYYGGNE---------IIDKSESLCQARALELYGLDPAKWGVNVQPLSGAPANLY : 133
PfSHMTc : HKRYYGGND---------YVDKIEELCYKRALEAFNVSEEEWGVNVQPLSGSAANVQ : 107
PfSHTMm : SIDLNRNIF---------VNNKNIENLEYIALNSFNLQKKYWGCLISNVSLNNNKSI : 113
            ##                                                   ##

             180         *       200         *       220
arabidC : VYTALLKPHERIMALDLPHGGHLSHGYQTDT---KKISAVSIFFETMPYRLDENTGY : 201
peaM    : VYTALLKPHDRIMALDLPHGGHLSHGYQTDT---KKISAVSIFFETMPYRLDESTGY : 210
humanC  : VYTALVEPHGRIMGLDLPDGGHLTHGFMTDK---KKISATSIFFESMPYKVNPDTGY : 180
humanM  : VYTALLQPHDRIMGLDLPDGGHLTHGYMSDV---KRISATSIFFESMPYKLNPKTGL : 203
yeastC  : VYQAIMKPHERLMGLYLPDGGHLSHGYATEN---RKISAVSTYFESFPYRVNPETGI : 171
yeastM  : VYSAIMNVGERLMGLDLPDGGHLSHGYQLKSG--TPISFISKYFQSMPYHVDHTTGL : 188
PfSHMTc : ALYALVGVKGKIMGMHLCSGGHLTHGFFDEK---KKVSITSDLFESKLYKCNSE-GY : 160
PfSHTMm : DDYFFIKLYGHFLKKE-CKI--LRINYCLEQNIENNVSNDIMQNLYNIININKNRN-E : 166
                                #    ##&            #

      *       240          *       260          *       280
arabidC : IDYDQLEKSAVLFRPKLIVAGASAYARLYDYARIRKVCN-KQK-----AVMLADMAH : 252
peaM    : IDYDQLEKSATLFRPKLIVAGASAYARLYDYARIRKVCD-KQK-----AVLLADMAH : 261
humanC  : INYDQLEENARLFHPKLIIAGTSCYSRNLEYARLRKIAD-ENG-----AYLMADMAH : 231
humanM  : IDYNQLALTARLFRPRLIIAGTSAYARLIDYARMREVCD-EVK-----AHLLADMAH : 254
yeastC  : IDYDTLEKNAILYRPKVLVAGTSAYCRLIDYKRMREIAD-KCG-----AYLMVDMAH : 222
yeastM  : IDYDNLQVLAKAFRPKVIVAGTSAYSRLIDYARFKEISQ-GCG-----AYLMSDMAH : 239
PfSHMTc : VDMESVRNLALSFQPKVIICGYTSYPRDIDYKGFREICD-EVN-----AYLFADISH : 211
PfSHTMm : PNYDEIQKISNDFNPDIIYFDESNNPYNIDYDRFIKGLKNKNNKIHNKPIIITNMNN : 223
                                  &                           (#)  #
```

Supp. Fig. 2 part (i)

```
                *         300         *        320         *        340
arabidC : ISGLVAAGVIPSPFEYADVVTTTTHKSLRGPRGAMIFFRKGLKEIN-KQGKEVMYDY : 308
peaM    : ISGLVAAGVIPSPFDYADVVTTTTHKSLRGPRGAMIFFRKGLKEVN-KQGKEVFYDY : 317
humanC  : ISGLVAAGVVPSPFEHCHVVTTTTHKTLRGCRAGMIFYRKGVKSVDPKTGKEILYNL : 288
humanM  : ISGLVAAKVIPSPFKHADIVTTTTHKTLRGARSGLIFYRKGVKAVDPKTGREIPYTF : 311
yeastC  : ISGLIAAGVIPSPFEYADIVTTTTHKSLRGPRGAMIFFRRGVRSINPKTGKEVLYDL : 279
yeastM  : ISGLVAANVVPSPFEHSDIVTTTTHKSLRGPRGAMIFFRKGIKSVTKK-GKEIPYEL : 295
PfSHMTc : ISSFVACNLLNNPFTYADVVTTTTHKILRGPRSALIFFNK-------K--RNP--GI : 257
PfSHTMm : KANLISQNLINSPFTHSDIVFTYFNENFRAHNSFVIFYKKGYKCVNTD-GHIIEYDY : 279
                                 # ##

                  *         360         *        380         *         40
arabidC : EDRINQAVFPGLQGGPHNHTITGLAVALKQARTPEYKAYQDQVLRNCSKFAELDIRP : 365
peaM    : EDKINQAVFPGLQGGPHNHTITGLAVALKQATTPEYRAYQEQVLSNSSKFAK----- : 369
humanC  : ESLINSAVFPGLQGGPHNHAIAGVAVALKQAMTLEFKVYQHQVVANCRALSE----- : 340
humanM  : EDRINFAVFPSLQGGPHNHAIAAVAVALKQACTPMFREYSLQVLKNARAMAD----- : 363
yeastC  : ENPINFSVFPGHQGGPHNHTIAALATALKQAATPEFKEYQTQVLKNAKALES----- : 331
yeastM  : EKKINFSVFPGHQGGPHNHTIGAMAVALKQAMSPEFKEYQQKIVDNSKWFAQ----- : 347
PfSHMTc : DQKINSSVFPSFQGGPHNNKIAAVACQLKEVNTPFFKEYTKQVLLNSKALAE----- : 309
PfSHTMm : EKKLKY-AFDDIYL---NNIFFSFFTSFKLMKNEEFKEYVKQIKENTYILYK----- : 327
                             #

          0         *         420         *        440         *
arabidC : TVIISYGLSMQTLLAKGYDLVSGGTDNHLVLVNLKNKGIDGSRVEKVLELVHIAANK : 422
peaM    : -ALS----------EKGYDLVSGGTENHLVLVNLKNKGIDGSRVEKVLELVHIAANK : 415
humanC  : -ALT----------ELGYKIVTGGSDNHLILVDLRSKGTDGGRAEKVLEACSIACNK : 386
humanM  : -ALL----------ERGYSLVSGGTDNHLVLVDLRPKGLDGARAERVLELVSITANK : 409
yeastC  : -EFK----------NLGYRLVSNGTDSHMVLVSLREKGVDGARVEYICEKINIALNK : 377
yeastM  : -ELT----------KMGYKLVSGGTDNHLIVIDLSGTQVDGARVETILSALNIAANK : 393
PfSHMTc : -CLL----------KRNLDLVTNGTDNHLIVVDLRKYNITGSKLQETCNAINIALNK : 355
PfSHTMm : --YI----------NRKYFHIQYSQNNSFFNLNPSSCTFNIQEFYLLCNKLNIYFD- : 371

           460         *         480         *         500         *
arabidC : NTVPGDVSAMVPGGIRMGTPALTSRGFIEEDFAKVAEYFDLAVKIALKIKAESQGIY : 479
peaM    : NTVPGDVSAMVPGGIRMGTPALTSRGFVEEDFVKVAEYFDAAVSLALKVKAES---- : 468
humanC  : NTCPGDRSALRPSGLRLGTPALTSRGLLEKDFQKVAHFIHRGIELTLQIQSDTG--- : 440
humanM  : NTCPGDRSAITPGGLRLGAPALTSRQFREDDFRRVVDFIDEGVNIGLEVKSKT---- : 462
yeastC  : NSIPGDKSALVPGGVRIGAPAMTTRGMGEEDFHRIVQYINKAVEFAQQVQQSLP--- : 431
yeastM  : NTIPGDKSALFPSGLRIGTPAMTTRGFGREEFSQVAKYIDSAVKLAENLKTLEPTT- : 449
PfSHMTc : NTIPSDVDCVSPSGIRIGTPALTTRGCKEKDMEFIADMLLKAILLTDELQQKYG--- : 409
PfSHTMm : --ILKDKSSNQKS-FNIGTNNLTSLGLLTHDIKNVAEFFNESVVLYFYLKEKSK--- : 422
          #                #

             520         *         540         *         560
arabidC : KKSFGTKLKDFVATMQSNEKLQSEMSKLREMVEEYAKQFPTIGFEKETMRYKE-  : 532
peaM    : K---GTKLKDFVEALQTSSYVQSEISKLKHDVEEFAKQFPTIGFEKATMKYNK-  : 518
humanC  : ---VRATLKEFKERLAGD-KYQAAVQALREEVESFASLFPLPGLPDF-------  : 483
humanM  : -----AKLQDFKSFLLKDSETSQRLANLRQRVEQFARAFPMPGFDEH-------  : 504
yeastC  : --KDACRLKDFKAKVDEG---SDVLNTWKKEIYDWAGEYPLAV-----------  : 469
yeastM  : KLDARSRLNEFKKLCNES---S-EVAALSGEISKWVGQYPVPGDI---------  : 490
PfSHMTc : -----KKLVDFKKGLVNN----PKIDELKKEVVQWAKNLPFA------------  : 442
PfSHTMm : -----LTNMSFIQYIEDN-SSASDIYSLAVDISSFISSYPSPYTNE--------  : 462
```

Supp. Fig. 2 part (ii)

Supplementary Figure 2 – Sequence alignments of the PfSHMT isoforms.

Alignment of PfSHMTc and PfSHMTm with cytoplasmic (C) and mitochondrial (M) SHMTs from other organisms, showing the absence in PfSHMTm of most of the highly conserved residues associated with the active site {Franca, 2005 #1015}. Ampersands (&) under the sequences indicate such residues that are conserved in PfSHMTm (4 in total), hashes (#) indicate such residues that are not conserved in PfSHMTc (16 in total) and ((#)) indicates the one conservative replacement.
